# Production and characterisation of a recombinant scFv reactive with human gastrointestinal carcinomas

**DOI:** 10.1038/sj.bjc.6600365

**Published:** 2002-08-12

**Authors:** D-J Kim, J-H Chung, Y-S Ryu, J-H Rhim, C-W Kim, Y Suh, H-K Chung

**Affiliations:** Department of Biochemistry and Molecular Biology, Cancer Research Institute, Seoul National University College of Medicine, Seoul, 110-799, Korea; Department of Pathology, Cancer Research Institute, Seoul National University College of Medicine, Seoul, 110-799, Korea

**Keywords:** SC142-reactive antigen, SC142 antibody, SC142 scFv, expression cloning

## Abstract

SC142-reactive antigen are highly glycosylated glycoproteins expressed on tissues of gastric and colon cancers but not on normal tissues. Murine SC142 antibody specific for the SC142-reactive antigen has been produced by immunisation with SNU16 stomach cancer cells. However, SC142 antibody has several potential problems such as high immunogenicity and poor tumour penetration owing to their large size. To improve tumour penetration potential *in vivo*, recombinant single-chain fragments have been produced using the original hybridoma cells as a source of variable heavy- and variable light-chain-encoding antibody genes. The use of the polymerase chain reaction, expression cloning technology and gene expression systems in *E. coli* has led to the production of SC142 single-chain fragments, which was similar in activity to the SC142 parent antibody confirmed by immunohistochemistry. Analysis by DNA sequencing, SDS–PAGE and Western blotting has demonstrated the integrity of the single-chain fragments. Competitive ELISA showed that SC142 single-chain fragments originated from parent SC142 antibody. BIAcore biosensor binding experiments showed that the SC142 single-chain fragments had an ideal dissociation rate constant as a tumour imaging reagent. These results illustrate the potential application of these novel products as an immunodiagnostic and further immunotherapeutic reagent.

*British Journal of Cancer* (2002) **87**, 405–413. doi:10.1038/sj.bjc.6600365 www.bjcancer.com

© 2002 Cancer Research UK

## 

Mucins are high-molecular-weight glycoproteins composed by a carbohydrate moiety (mainly O-glycans) that represents 50–80% of their total mass and peptide core; also called apomucin, it is rich in Thr and Ser (Verma and Davidson, 1994). A variety of alterations of mucins have been described in metaplastic and malignant disease of the stomach (Correa, 1988). To detect those cancer associated alterations of gastric mucin, a number of monoclonal antibodies (mAbs) were developed. Although their specificity for cancer was limited compared with that of genetic markers such as p53, APC, c-met, k-sam and c-erbB-2, immunohistological detection of antigen is much easier than molecular biological analysis of those gene abnormalities, and this is of practical importance.

In the previous study, we developed monoclonal antibodies elicited to the human stomach carcinoma cell line SNU16 to detect useful markers for gastric cancer. One of these monoclonal antibodies, SC142, detects an antigen present on adenocarcinoma cells of stomach. Preliminary studies on the molecular properties of the SC142-reactive antigen suggest that the epitope is sensitive to O-glycanase and is expressed on a large, heavily glycosylated molecule indicating that the antigen is probably mucin (Hong *et al*, 2001).

In immunohistochemical studies, SC142-reactive antigen was not detected in normal gastrointestinal epithelium, whereas it was highly expressed in 78% of gastric cancers (29 out of 37) and 87% of colon cancers (27 out of 31) indicating that SC142 antibody can be a valuable tool to detect gastrointestinal cancers.

However, the use of whole antibody for *in vivo* imaging of cancer targets may have limitations due to their large molecular weight, with subsequent slow tumour uptake and long serum half-life (Power *et al*, 2001). Recombinant single chain variable fragments (scFvs), in which the two variable domains are covalently joined via a flexible peptide linker have overcome many of these problems. ScFvs usually show the same binding specificity and affinity as the monomeric form of the parent antibody (Bedzyk *et al*, 1990; Pantoliano *et al*, 1991) and may penetrate tumours more rapidly and evenly due to their relatively small size (*M_r_* 27 000 *vs* *M_r_* 50 000 to 900 000 for other immunoglobulin forms). The present investigation was initiated to produce derivatives of SC142 antibody that would improve tumour penetration potential *in vivo*. Here, we describe the production of a recombinant antibody fragment based on the variable region of the SC142 monoclonal antibody using expression cloning without antigen. Detailed analyses of the scFv confirm that its antigen recognition characteristics are similar to those of the parent antibody so that further diagnostic and therapeutic applications may be considered.

## MATERIALS AND METHODS

### RNA extraction

SC142 hybridoma cells (1×10^7^) were harvested by centrifugation (1000 **g** for 5 min), the supernatant removed by gentle aspiration, and the cellular pellet was vortexed briefly. Total RNA was extracted from the pellet using an Ultraspec RNA kit I (Biotecx, South Loop East, Houston). The RNA extract was then dissolved in 100 μl of sterile water, quantified, and assessed for purity by absorbance determination at 280 and 260 nm (Sambrook *et al*, 1989). Samples were stored at −70°C.

### ScFv assembly

**Figure 1:**
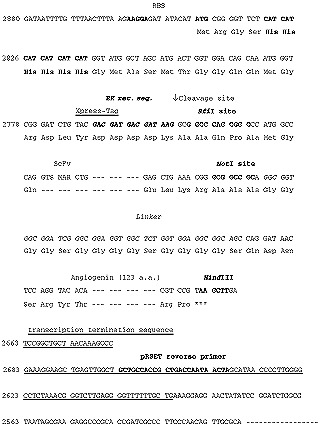
Relevant parts of the nucleotide and amino acid sequences of the SC142 scFv unit in pRSET-Angiogenin *Sfi*I/*Not*I vector. The pRSET *Sfi*I/*Not*I vector (Yi *et al*, 1999) was amplified with the primers as described in Materials and Methods. The vector was modified to include a *Hind*III site and angiogenin site after *Not*I site. The HIS-tag and Xpress-tag upstream from the enterokinase cleavage site can be removed by enterokinase treatment. The numbers in the left column indicate the nucleotide numbers of pRSET B (Invitrogen).

Hybridoma total RNA served as the template for constructing SC142 scFv using the Recombinant Phage Antibody System (RPAS; Pharmacia, Uppsala, Sweden); incorporating many of the features described by Mccafferty *et al* (1990) and Winter and Milstein (1991). Isolated, agarose gel-purified VH- and VL-encoding DNA were subsequently spliced together by PCR using primers designed to introduce a linking sequence between the two gene segments and specific restriction sites at both 5′ (*Sfi*I) and 3′ (*Not*I) ends of the spliced sequence. Restriction digestion with *Sfi*I and *Not*I endonucleases, and agarose gel purification of the digested linked product, preceded ligation of this DNA into the *Sfi*I- and *Not*I-digested pRSET-Angiogenin *Sfi*I/*Not*I vector (Figure 1

). This vector was constructed from the pRSET *Sfi*I/*Not*I vector (Yi *et al*, 1999) by PCR. The PCR protocol used the forward primer 5′-GTTATCCT GGCT GCCGCCTCCACCAGAGCCACCTCCGCCCGATCCGCCACCGCCTGCGGCCGCCCG-3′ and the back primer 5′-CGTCCGTAAGCTTGATCCGGCTGCTAACAAAGCC-3′ for 30 cycles of 1 min at 95°C, 2 min at 55°C, and 1 min at 72°C. After 1.0% agarose gel electrophoresis of the PCR products, the correctly sized band of 3.0 kb was excised. The DNA was purified using the Qiaex II gel extraction kit (Qiagen, Stanford Valencia, CA, USA). Human angiogenin was linked to equal moles of the vector by overlap extension PCR with the forward primer 5′-TCAAGCTTACGGACG-3′ and the back primer 5′-TAAGCTTGATCCGGCTGCTAACAAAGCC-3′. PCR reagents were subjected to 30 cycles of 1 min at 95°C, 2 min at 55°C, and 1 min at 72°C. After 1.0% agarose gel electrophoresis of the PCR products, the correctly sized band of 3.3 kb was excised. This vector was digested with *Hind*III restriction enzyme and ligated using a ligation kit (Gibco-BRL, Grand Island, NY, USA). This vector was amplified in heat competent *E. coli* XL-1 blue cells (Stratagene, La Jolla, CA, USA) and then extracted with a QIAprep spin miniprep kit (Qiagen). This vector was used to transform competent BL21 *E. coli* cells (Novagen, Madison, WI, USA) using a heat shock (42°C) transformation method. Ten individual colonies were selected from each transformation. Transformed BL21 *E. coli* were then subjected to culture protocol, producing a recombinant scFv of SC142 encoding VH- and VL-linked genes.

### Primer design

The primers used in the scFv assembly were provided in the RPAS mouse scFv module. Primers used in sequencing reactions and in PCR analyses were T7 primers.

### Analysis of scFv clones by sodium dodecyl sulphate (SDS) polyacrylamide gel electrophoresis (SDS–PAGE) and Western blotting

Induction of scFv expression was achieved using the *Lac*Z promoter of the pRSET-Angiogenin *Sfi*I/*Not*I vector by the addition of isopropyl beta-D-thiogalactopyranoside (IPTG) substrate into LB medium supplemented with 100 μg ml^−1^ ampicillin (Sambrook *et al*, 1989). A total of 10 ml of substrate was added. Ten clones were then chosen and subjected to scFv expression for functional immunoglobulin gene analysis before SDS–PAGE and Western blot analyses were performed.

The cells harbouring expressed scFv were solubilised with SDS-loading buffer (bromophenol blue (0.05% w v^−1^), glycerol (25%)). Proteins were separated by proper concentration of SDS–PAGE and stained with Coomassie brilliant blue R (Sigma, St. Louis, MO, USA) or transferred to nitrocellulose membranes (Schleicher & Schuell, Keene, NH, USA). Membranes were incubated for 1 h with the blocking buffer (5% non-fat milk (w v^−1^) in phosphate-buffered saline (PBS, pH 7.4) containing 0.2% (w v^−1^) Tween 20 (PBS/Tween) for Western blot analysis. Anti-Xpress Abs were used at a 1 : 1000 dilution, and secondary horseradish peroxidase-conjugated Abs (Pierce, Rockford, IL, USA) at a 1 : 10000 dilution.

The SC142 scFv gene fragment, a fusion protein of predicted size in SDS–PAGE and Western blot analyses and found to be positive in ELISA and confirmed by DNA sequencing, was subcloned into pRSET *Sfi*I/*Not*I vector (Yi *et al*, 1999) and transformed into *E. coli* BL21 (DE3) cells for SC142 scFv purification.

### DNA sequencing analysis

The clone containing SC142-encoding DNA, and in which expression of the scFv was demonstrated using Western blotting, was further analysed using manual Sanger dideoxy DNA sequencing.

### Production of SC142 scFv

Expression of the SC142 scFv fragment was induced via the addition of 1 mM IPTG followed by incubation for 3 h at 37°C. From 200 ml culture, the cells were harvested, washed twice with PBS and the final cell pellet was stored at −20°C prior to scFv purification.

### Purification of SC142 scFv

To purify scFv, the washed cell pellet was resuspended in PPET buffer containing 2 mM EDTA, 2% Triton X-100, and 1 mM PMSF in PBS, ultrasonicated and centrifuged at 24 300 **g** for 30 min. The supernatant was removed and the pellet was resuspended in fresh PPET buffer. This step was repeated four times. The final supernatant was discarded and the inclusion body pellet was subjected to SDS–PAGE, Coomassie brilliant blue staining and Western blotting for check of purity.

### Refolding of SC142 scFv

The inclusion body pellet was solubilised in 50 mM Tris-HCl (pH 8.0) containing 6 M guanidine-HCl (GuHCl), 200 mM NaCl and 10 mM 2-mercaptoethanol (β-ME) overnight at 4°C. The solubilised precipitant was subsequently centrifuged at 12 000 r.p.m. and the supernatant was subjected to the refolding step. Refolding of scFv was performed according to Tsumoto *et al* (1998).

### Size-exclusion FPLC chromatography and molecular mass determination

Refolded scFv was analysed by size-exclusion chromatography on a calibrated Bio-Prep SE-100/17 column (Bio-Rad Laboratories, Sydney, Australia) at a flow rate of 0.5 ml min^−1^ in 50 mM Tris (pH 8.0). The column was calibrated using standard molecular mass markers, containing thyroglobulin, IgG, ovalbumin, myoglobin, vitamin B_12_ (Bio-Rad Laboratories, Sydney, Australia).

### Purification of SC142-reactive antigen

Culture supernatants and membrane fractions of SNU16 cells were used for cesium chloride density gradient ultracentrifugation performed using the method described by Creeth and Denborough (1970). The densities of every fraction was determined by weighing 200 μl in a calibrated micropipette. In order to identify the fractions containing the SC142-reactive antigen, duplicates of 100 μl from each fraction were then applied to enzyme-linked immunosorbent assay described by Ching and Rhodes (1990). CsCl was removed by dialysis against water, and assays of each fraction's protein concentration was performed.

### Biotinylation of SC142 scFv

Recombinant SC142 scFv was coupled with biotin at concentrations of 100 μg ml^−1^ in PBS by the N-hydroxysuccinimide (NHS) esters coupling procedure described by the manufacturer (Pierce). Similarly, the degree of biotin incorporation was determined by the HABA method recommended by the manufacturer.

### Immunoreactivity by enzyme-linked immunosorbent assay (ELISA) and competitive ELISA

Microtitre plates (96-well, flat bottomed, Nunc) were coated with purified SC142-reactive antigen (5 μg ml^−1^) for 16 h at 4°C. The wells were washed four times with PBS/Tween, and the remaining non-specific sites were blocked by the addition of 3% BSA for 1 h. After four washes in PBS/Tween, 50 μl recombinant SC142 scFv (25 μg ml^−1^) was added to each well and incubated for 8 h. For the competition ELISA, 25 μl of recombinant SC142 scFv (50 μg ml^−1^), and 25 μl of SC142 monoclonal antibody (50 μg ml^−1^) in 3% BSA/PBS, were added to each well and incubated for 8 h. The control well was incubated with only the same amount of recombinant SC142 scFv. Another control well was incubated with only 50 μl of SC142 monoclonal antibody and developed with goat anti-mouse IgGAM/horseradish peroxidase conjugate at 1 : 1000 in 3% BSA/PBS. The plates were then washed ten times and 50 μl of anti-Xpress antibody at 1 : 1000 in 3% BSA/PBS was added, to each well, and incubated for 1 h. The plates were washed ten times in PBS/Tween. And then 50 μl of goat anti-mouse IgG/horseradish peroxidase conjugate at 1 : 1000 in 3% BSA/PBS was added, to each well, and incubated for 1 h. After incubation the plates were washed 10 times in PBS/Tween. In addition to a solution of 0.1 M citrate phosphate buffer pH 5.8, with 33% (v v^−1^) H_2_O_2_ (Sigma) which was added at a concentration of 0.3 μl ml^−1^, 2 mg ml^−1^ of *o*-phenylenediamine (Sigma) was added to each of the wells at 100 μl per well. Analysis of colour development was assessed over a 10 min period using an ELISA reader.

### Surface plasmon resonance studies

The BIAcore biosensor (Pharmacia), which uses surface plasmon resonance detection and permits real-time kinetic analysis of two interacting species, was used to measure the binding kinetics of the SC142 antibody and scFv. Covalent immobilisation of SC142 antibody and recombinant SC142 scFv was performed via free amine groups using N-hydroxysuccinimide/N-ethyl-N′ (dimethylaminopropyl) carbodiimide (NHS/EDC) coupling as described previously (Gruen *et al*, 1993). Immobilisation of SC142 monoclonal antibody was performed in 10 mM sodium acetate, pH 5.0, at a flow of 5 μl min^−1^ (6066 resonance units (RU) immobilised). Similarly, immobilisation of SC142 scFv was performed in 10 mM sodium acetate, pH 4.8, with an initial injection of 100 μl at 5 μl min^−1^ to immobilise 8186 RU. All binding experiments were performed on an upgraded Pharmacia BIAcore 2000 in HBS buffer, pH 7.4 (10 mM HEPES, 150 mM NaCl, 3.4 mM EDTA) including 0.005% (v v) of the nonionic detergent P20 (Pharmacia). The kinetic constants for dissociation (*K*_D_) were evaluated using BIAevaluation 3.0 software (Pharmacia), from average *k_a_* (association rate) and *k_d_* (dissociation rate) kinetics.

### Immunohistochemistry

Immunohistochemistry was performed using the method described by Denton *et al* (1997). The biotin-labelled SC142 scFv was applied for 1 h. Optimal antibody concentrations were determined by titration. To detect scFv binding, specimens were incubated for 1 h with streptavidin/horseradish peroxidase conjugate (1 : 50).

## RESULTS

### SC142 scFv assembly and expression

**Figure 2:**
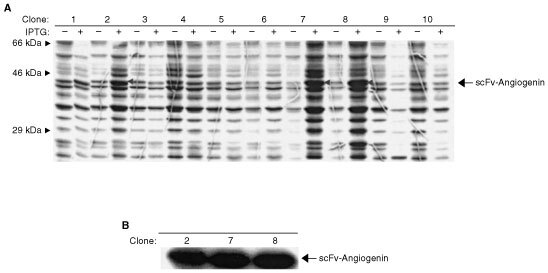
Screening of scFv by scFv expression. *E. coli* BL21 transformed cells containing expressed scFv were lysed and subjected to SDS–PAGE (12%) and stained with Coomassie blue staining after separating proteins on reducing SDS–PAGE gels. (**A**) Protein expression pattern before and after IPTG induction. Left-oriented arrowheads indicate relatively highly expressed protein bands. (**B**) Western blot analysis of selected clones using anti-Xpress antibody.

Insertion of SC142 VH- and VL-specific DNA in the scFv assembly process was analysed by expression of the DNA from transformants of BL21 *E. coli* clones grown on ampicillin plates. These tests were performed using Coomassie blue staining and Western blot analysis with anti-Xpress antibody. The pRSET-Angiogenin *Sfi*I/*Not*I expression vector uses a reporter sequence known as the Xpress-tag to show the presence of scFv (Figure 1). A murine antibody recognising the Xpress-tag peptide sequence allows the presence of expressed scFv to be visualised on Western blots. The use of this reporter, plus analysis of the product size through SDS–PAGE, reveals the presence of intact recombinant scFv. Of 10 randomly selected clones, three produced expression products of approximately 45 kDa in length (Figure 2

). The size of these products is consistent with the assembly of linked VH and VL into the vector containing human angiogenin. To verify whether choosing clones by product size was suitable for selecting clones with functional antibodies, we performed ELISA on the clones containing fragments of the correct size. The results of the ELISA demonstrated that all of the clones with correctly sized fragments were functional. All of the functional clones were further analysed by employing DNA sequencing techniques using the primers T7 to identify the exact DNA sequence of the inserted SC142-encoding region. DNA sequencing analysis of selected functional clones demonstrated that all of the functional clones were identical and that mutation of the linker sequence, in which Gly^120^ is changed to Cys and Ser^129^, has changed to Pro (Table 1

**Table 1:**
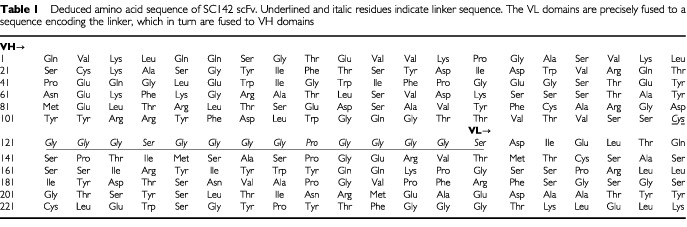
Deduced amino acid sequence of SC142 scFv. Underlined and italic residues indicate linker sequence. The VL domains are precisely fused to a sequence encoding the linker, which in turn are fused to VH domains

). Although mutation of the linker sequence occurred, because binding activity of all the clones was not changed through ELISA, we analysed the characteristics of SC142 scFv further.

**Figure 3:**
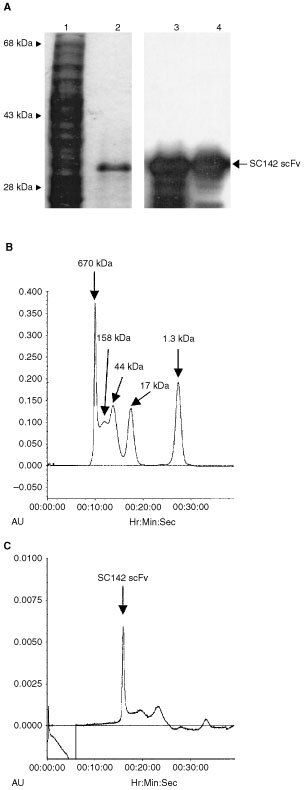
SDS–PAGE and Western blot analysis of purified SC142 scFv. Left panel of (**A**) is Coomassie blue stained SDS–PAGE and right panel of (**A**) is Western blot analysis of *E. coli* BL21 (DE3) cells overproducing SC142 scFv probed with anti-Xpress antibody. Lanes 1 and 3 are whole cell lysate and lane 2 and 4 are purified SC142 scFv. (**B**) chromatogram of molecular standards; (**C**) chromatogram of the purified and refolded SC142 scFv.

For this additional characterisation of SC142 scFv, we subcloned the scFv gene fragments into the pRSET *Sfi*I/*Not*I vector, transformed it into *E. coli* BL21 cells, and produced SC142 scFv. Preliminary studies were performed in which whole-cell extracts, periplasmic extracts, and supernatants from cultures of clones were tested for the presence of scFv by SDS–PAGE and Western blotting. All of the clones expressed insoluble recombinant scFv in sufficient quantities to be revealed in the inclusion bodies by Western blotting (data not shown). Inclusion bodies were solubilised and renatured as described in Materials and Methods. Appropriate fractions were further analysed by SDS–PAGE and Western blotting. Figure 3A

(lane 2 in the left panel) depicts the purified protein as a single band of approximately 30 kDa by SDS–PAGE. After Western blot analysis of the same sample, Figure 3A (lane 4 in the right panel) shows that this protein contains the Xpress-tag sequence and therefore identify it as SC142 scFv.

The monomeric state of SC142 scFv was analysed by gel filtration. Purified SC142 scFv contained predominantly a peak eluting with an apparent molecular mass of ∼30 kDa, consistent with a monomeric scFv with a calculated *M_r_* of 30 256 (Figure 3C). This result confirmed that the SC142 scFv with a 15 amino acid residue linker joining the VH and VL domains formed a stable scFv, not multimers.

### Purification of SC142-reactive antigen

**Figure 4:**
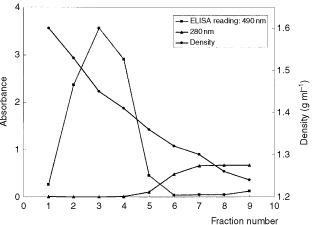
Purification of SC142-reactive antigen by CsCl density gradient centrifugation. Enzyme-linked SC142 antibody assay of the fractions were separated by cesium chloride density gradient ultracentrifugation showing a major peak with density >1.40 g ml^−1^.

To characterise recombinant SC142 scFv, we needed to purify the SC142-reactive antigen, a novel tumour-associated mucin. Given that immunoaffinity chromatography was an ineffective means of purifying the SC142-reactive antigen, alternative purification methods were sought. Typically non-glycosylated proteins and glycoproteins have densities ranging from 1.20 to 1.30 g ml^−1^, whereas heavily glycosylated glycoproteins, such as mucin, have densities in excess of 1.40 g ml^−1^ (Lan *et al*, 1985). The SC142-reactive antigen is expressed in SNU16 cells, and also secreted into culture medium of SNU16 cells. When the culture supernatants or membrane fractions of SNU16 cells were subjected to cesium chloride density gradient centrifugation, a prominent peak of SC142 antibody binding glycoprotein sedimented with a density of more than 1.40 g ml^−1^ as would be expected for a mucin (Figure 4

). Table 2

**Table 2:**
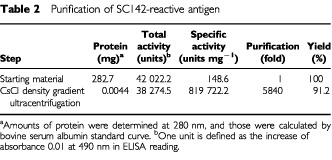
Purification of SC142-reactive antigen

summarises the purification of SC142-reactive antigen.

### Competitive ELISA

**Figure 5:**
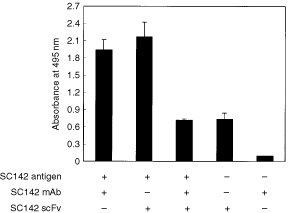
SC142 scFv binds specifically to SC142-reactive antigen confirmed by competition of the SC142 scFv with SC142 mAb to SC142-reactive antigen. Bound scFv was detected with anti-Xpress antibody. The vertical bars are propotional to the absorbance obtained by ELISA, and bars represent the standard deviations.

To confirm whether SC142 scFv originates from parental SC142 antibody, we performed competitive ELISA. A checkerboard assay was used before competitive ELISA to optimise the concentrations of both SC142-reactive antigen and SC142 scFv. The coating concentration of SC142-reactive antigen was 5 μg well^−1^ and the concentration of SC142 scFv was 25 μg well^−1^ (data not shown). As the result of competitive ELISA, SC142 scFv binding was completely inhibited by the original SC142 antibody (Figure 5

).

### BIAcore analysis

**Figure 6:**
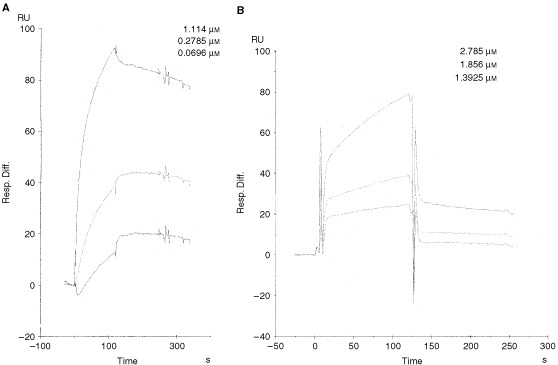
Sensorgrams showing apparent kinetic constants for the binding of SC142-reactive antigen to immobilised antigen binding fragments determined in the BIAcore. (**A**) SC142 antibody binding to SC142-reactive antigen; (**B**) SC142 scFv binding to SC142-reactive antigen. The surface was regenerated with 30 μl of 1 M NaCl in 10 mM glycine (pH 1.85). The data was evaluated with the 1 : 1 Langmuir binding model to estimated the kinetic and binding constants.

Generally, scFvs have the same binding affinity as the monomeric form of the parent antibody, but this is not true in all cases. Although SC142 scFv originated from the SC142 antibody and demonstrated the same binding specificity as the SC142 antibody, its binding affinity could nonetheless differ from that of the SC142 antibody. BIAcore analysis was therefore used to determine an exact affinity constant for both SC142 scFv and the SC142 antibody (Figure 6

, see Materials and Methods). SC142 scFv exhibited a relative *K*_D_ of 6.68×10^−7^ M (Table 3

**Table 3:**
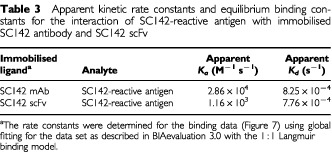
Apparent kinetic rate constants and equilibrium binding constants for the interaction of SC142-reactive antigen with immobilised SC142 antibody and SC142 scFv

). This value compares favourably with affinity constants for the intact SC142 monoclonal antibody (2.88×10^−8^ M).

### Immunohistochemistry

**Figure 7:**
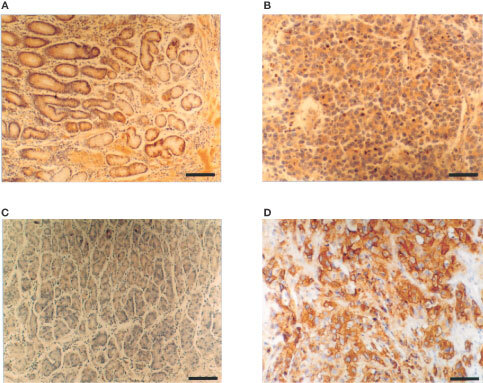
Binding of SC142 scFv to normal stomach tissue (**C**) and poorly differentiated human gastric adenocarcinoma tissue (**D**) as detected by immunohistochemical staining comparable with binding of SC142 antibody to normal stomach tissue (**A**) and poorly differentiated human gastric adenocarcinoma tissue (**B**). (**A**) and (**C**) *Scale bar*=60 μm; (**C**) and (**D**) *Scale bar*=30 μm. To detect scFv, the system of biotin-labelled SC142 scFv and streptavidin/horseradish peroxidase conjugate was used as this system eliminates nonspecific binding signal because the covalent-like interaction between biotin and streptavidin is so strong that streptavidin is highly specific for biotin. SC142 scFv was biotinylated as described in Materials and Methods.

Immunohistochemical staining was used to test the ability of SC142 scFv to bind to tumour cells. It shows the ability of SC142 antibody (Figure 7B

) and SC142 scFv (Figure 7D) to preferentially bind to stomach tumour tissue sections. The surface staining pattern is characteristic of the expression of SC142-reactive antigen. Some weak staining to the normal cells using the whole antibody is seen in Figure 7A but is not observed for the scFv (Figure 7C).

## DISCUSSION

Four decades of research show that the immune response to tumour antigens and probably to other antigens, is diminished in patients with cancer. In addition a widely quoted article in this journal reported no evidence of immune response to 27 different spontaneous tumours in mice (Hewitt *et al*, 1976). Attempts for generating human antibodies against tumour antigen have been hampered by these reasons. Based on these facts, immune mice would be an alternative and attractive source to produce antibodies specific for tumour antigen.

Over the past years, we have generated antibodies specific for stomach cancer-associated antigen by immunisation with the SNU-16 stomach cancer cell line, and SC142 antibody is one of them (Hong *et al*, 2001). The SC142 monoclonal antibody, IgM, recognised O-glycan in a mucin-like glycoprotein. The SC142-reactive antigen was expressed mainly in gastrointestinal cancers, especially in colon and stomach cancers. Practically, all normal gastrointestinal tissues did not react with SC142 antibody. However, SC142 antibody has high molecular weight thereby limiting their potential use for *in vivo* imaging applications. In order for whole antibodies against tumour to be used as diagnostic imaging reagents, they have been minimised to recombinant antibodies. Also, conversion of the hybridoma-produced antibody to a bacterially expressed fragment is a prerequisite of modelling and humanisation for a further therapeutic reagent (Hoogenboom *et al*, 1998).

The results presented here confirm the successful production of a recombinant single-chain antibody fragment (scFv) retaining the capacity to bind to the SC142-reactive antigen. Assembly of scFv, using the antibody variable region genes and the fusion protein expression cloning system, produces a recombinant protein in which the structural features that define the high specificity of the parent antibody for its antigen are retained. Alterations in the sequence of SC142 scFv probably arose from unspecific priming under the amplification procedure. Although prolines in the linker regions of scFv may not be the most optimal amino acid residues for a flexible linker, they did not influence on function of the scFv.

The fusion protein expression cloning system was chosen because of two reasons. Firstly, it was difficult to purify SC142-reactive antigen required for constructing a phage display library and producing scFvs. SC142-reactive antigen purification by immunoaffinity column chromatography was unsuccessful, since the SC142 antibody shows poor antigen binding when coupled to insoluble matrix. Therefore, we needed the system developing recombinant SC142 scFv without antigen. Secondly, only a few percentage of the clones produced antibodies in the expected size range in establishing antibody libraries from lymphocytes or hybridomas because there are aberrant immunoglobulin genes that carry the nonsense mutation in the mRNA pool of a lymphocyte which has one functional immunoglobulin gene. Therefore, we used the fusion protein expression cloning system to overcome aberrant mRNA contamination when cloning functional immunoglobulin RNA. The methodology we have selected here, based on fusion protein expression cloning, enabled us to distinguish functional immunoglobulin genes that produced protein products of predicted size from aberrant immunoglobulin genes.

BIAcore assays indicate recombinant SC142 scFv is high-affinity anticarbohydrate antibody. The reason that *k_on_* and *k_off_* of SC142 scFv are different from those of the original antibody may result from heterogeneity of functional light chain gene from single B lymphocytes (Jena *et al*, 2000). Therefore, binding affinity of SC142 scFv is slightly different from the original SC142 antibody. Also, BIAcore data is consistent with competition ELISA data because BIAcore assays show that on-rate of SC142 antibody is much faster than that of SC142 scFv and that half life of SC142 antibody-SC142-reactive antigen is theoretically about 16 h. For this reason SC142 scFv binding is inhibited completely by SC142 antibody during competition ELISA.

The scFv format was chosen for two reasons. First, scFvs are advantageous for the treatment of solid cancers. Because of their small size, these proteins penetrate faster and deeper into tissues and clear more rapidly from the blood than whole IgG or Fabs (Mao *et al*, 1999). Also, the lack of constant regions mitigates against retention by Fc receptors found in most tissues and organs, which further reduces their side effects (Yamaguchi *et al*, 1998). The rapid blood clearance and good tumour penetration of scFvs offer potential advantages over larger antibody molecules for cancer therapy such as radioimmunoguided surgery and antibody-directed enzyme-prodrug therapy (Wels *et al*, 1992; Mayer *et al*, 2000). Also, these characteristics of scFv are applicable to cancer imaging. Cancer imaging requires small, fast penetrating but tightly binding targeting modules, with a rapid plasma clearance. Often scFv fragments fail on just one parameter, i.e., their off-rate of binding antigen is too fast. This results in insufficient tumour-uptake and poor imaging. Most monoclonal antibodies and also most antibodies from primary phage libraries have typical off-rates that are not better than 10^−4^ s^−1^ at best. With an off-rate of 10^−4^ s^−1^, the half life of the antibody-antigen complex is theoretically 1.9 h. A seven-fold improvement, to the 7×10^−4^ s^−1^ range, would drive to an antigen-antibody half life of 13.3 h. BIAcore assays show that SC142 scFv has an ideal off-rate constant and SC142 scFv could be a good tumour imaging reagent.

Second, because factors likely to influence immunogenicity of therapeutic antibodies are murine constant regions, method of administration, patients' disease status, specificity of antibody, complement activation by antibody, and Fc receptor binding by antibody, scFvs have low immunogenic potential *in vivo* (Clark, 2000). Despite this advantage, scFvs can not trigger the appropriate human effector systems of complement and Fc receptors *in vivo* due to the absence of constant regions. To overcome this problem, scFvs can be designed to be more cytotoxic by attaching radioisotopes, cytotoxic drugs or protein drugs in cancer immunotherapy.

In summary, this is the first time a recombinant antibody against SC142-reactive antigen has been developed which is expressed in a high percentage of the cases of gastrointestinal cancer, and retains the ability to bind to tumour cells not normal cells. It may further prove to be a valuable tool in detecting useful markers for gastrointestinal cancer and in enhancing treatment of gastrointestinal cancer.

## References

[bib1] BedzykWDWeidnerKMDenzinLKJohnsonLSHardmanKDPantolianoMWAselEDVossJrEW1990 Immunological and structural characterization of a high affinity anti-fluorescein single-chain antibody J Biol Chem 265301861518620 2211723

[bib2] ChingCKRhodesJM1990 Purification and characterization of a peanut-agglutinin-binding pancreatic-cancer-related serum mucus glycoprotein Int J Cancer 45610221027 2351483 10.1002/ijc.2910450607

[bib3] ClarkM2000 Antibody humanization: a case of the ‘Emperor's new clothes’ Immunol Today 218397402 10916143 10.1016/s0167-5699(00)01680-7

[bib4] CorreaP1988 A human model of gastric carcinogenesis Cancer Res 48 35543560 3288329

[bib5] CreethJMDenboroughMA1970 The use of equilibrium-density-gradient methods for the preparation and characterization of blood-group-specific glycoproteins Biochem J 1175879891 5451909 10.1042/bj1170879 PMC1179046

[bib6] DentonGSekowskiMSpencerDIHughesODMurrayADenleyHTendlerSJPriceMR1997 Production and characterization of a recombinant anti-MUC1 scFv reactive with human carcinomas Br J Cancer 765614621 9303360 10.1038/bjc.1997.434 PMC2228010

[bib7] GruenLCKorttAANiceE1993 Determination of relative binding affinity of influenza virus N9 sialidases with the Fab fragment of monoclonal antibody NC41 using biosensor technology Eur J Biochem 2171319325 8223570 10.1111/j.1432-1033.1993.tb18249.x

[bib8] HewittHBBlakeERWalderAS1976 A critique of the evidence for active host defence against cancer, based on personal studies of 27 murine tumours of spontaneous origin Br J Cancer 333241259 773395 10.1038/bjc.1976.37 PMC2024987

[bib9] HongKMJangSJKongGSongKYParkJGKimDJChungJHLeeJHPaikMKChungHK2001 A new tumor-associated antigen of gastrointestinal carcinoma defined by monoclonal antibody SC142 J Cancer Res Clin Oncology 127 551558 10.1007/s004320100258 PMC12164805 11570576

[bib10] HoogenboomHRHenderikxPde HaardH1998 Creating and engineering human antibodies for immunotherapy Adv Drug Deliv Rev 311–2531 10837615 10.1016/s0169-409x(97)00091-4

[bib11] JenaPKLiuAHSmithDSAviszusKWysockiLJ2000 Sequence heterogeneity in Ig kappa transcripts from single B lymphocytes Mol Immunol 376265272 11000400 10.1016/s0161-5890(00)00055-9

[bib12] LanMSFinnOJFernstenPDMetzgarRS1985 Isolation and properties of a human pancreatic adenocarcinoma-associated antigen, DU-PAN-2 Cancer Res 451305310 3965141

[bib13] MaoSGaoCLoCHWirschingPWongCHJandaKD1999 Phage-display library selection of high-affinity human single-chain antibodies to tumor-associated carbohydrate antigens sialyl Lewisx and Lewisx Proc Natl Acad Sci USA 961269536958 10359820 10.1073/pnas.96.12.6953 PMC22023

[bib14] MayerATsiompanouEO'MalleyDBoxerGMBhatiaJFlynnAAChesterKADavidsonBRLewisAAWinsletMCDhillonAPHilsonAJBegentRH2000 Radioimmunoguided surgery in colorectal cancer using a genetically engineered anti-CEA single-chain Fv antibody Clin Cancer Res 6517111719 10815889

[bib15] McCaffertyJGriffithsADWinterGChiswellDJ1990 Phage antibodies: filamentous phage displaying antibody variable domains Nature 3486301552554 2247164 10.1038/348552a0

[bib16] PantolianoMWBirdREJohnsonSAselEDDoddSWWoodJFHardmanKD1991 Conformational stability, folding, and ligand-binding affinity of single-chain Fv immunoglobulin fragments expressed in *Escherichia coli* Biochemistry 30421011710125 1931943 10.1021/bi00106a007

[bib17] PowerBECaineJMBurnsJEShapiraDRHattarkiMKTahtisKLeeFTSmythFEScottAMKorttAAHudsonPJ2001 Construction, expression and characterisation of a single-chain diabody derived from a humanised anti-Lewis Y cancer targeting antibody using a heat-inducible bacterial secretion vector Cancer Immunol Immunother 505241250 11499807 10.1007/s002620100192 PMC11036816

[bib18] SambrookJFritschEFManiatisT1989 Molecular Cloning: a Laboratory Manual2nd ednCold Spring Harbor, New York: Cold Spring Harbor Press

[bib19] TsumotoKShinokiKKondoHUchikawaMJujiTKumagaiI1998 Highly efficient recovery of functional single-chain Fv fragments from inclusion bodies overexpressed in *Escherichia coli* by controlled introduction of oxidizing reagent–application to a human single-chain Fv fragment J Immunol Methods 2191–2119129 9831393 10.1016/s0022-1759(98)00127-6

[bib20] VermaMDavidsonEA1994 Mucin genes: structure, expression and regulation Glycoconj J 11 172179 7841791 10.1007/BF00731215

[bib21] WelsWHarwerthIMMuellerMGronerBHynesNE1992 Selective inhibition of tumor cell growth by a recombinant single-chain antibody-toxin specific for the erbB-2 receptor Cancer Res 522263106317 1358432

[bib22] WinterGMilsteinC1991 Man made antibodies Nature 349 293299 1987490 10.1038/349293a0

[bib23] YamaguchiADingKMaeharaMGoiTNakagawaraG1998 Expression of nm23-H1 gene and Sialyl Lewis X antigen in breast cancer Oncology 554357362 9663428 10.1159/000011878

[bib24] YiKChungJKimHKimIJungHKimJChoiISuhPChungH1999 Expression and characterization of anti-NCA-95 scFv (CEA 79 scFv) in a prokaryotic expression vector modified to contain a *Sfi*I and *Not*I site Hybridoma 183243249 10475238 10.1089/027245799315899

